# Semiconductor Gas Sensors: Materials, Technology, Design, and Application

**DOI:** 10.3390/s20226694

**Published:** 2020-11-23

**Authors:** Maria Vesna Nikolic, Vladimir Milovanovic, Zorka Z. Vasiljevic, Zoran Stamenkovic

**Affiliations:** 1 Institute for Multidisciplinary Research, University of Belgrade, 11030 Belgrade, Serbia; mariavesna@imsi.rs (M.V.N.); zorkav@imsi.rs (Z.Z.V.); 2 Faculty of Engineering, University of Kragujevac, 34000 Kragujevac, Serbia; vlada@kg.ac.rs; 3 IHP—Leibniz-Institut Für Innovative Mikroelektronik, 15236 Frankfurt (Oder), Germany

**Keywords:** semiconductor gas sensors, gas sensing materials, sensing technology, gas sensor applications

## Abstract

This paper presents an overview of semiconductor materials used in gas sensors, their technology, design, and application. Semiconductor materials include metal oxides, conducting polymers, carbon nanotubes, and 2D materials. Metal oxides are most often the first choice due to their ease of fabrication, low cost, high sensitivity, and stability. Some of their disadvantages are low selectivity and high operating temperature. Conducting polymers have the advantage of a low operating temperature and can detect many organic vapors. They are flexible but affected by humidity. Carbon nanotubes are chemically and mechanically stable and are sensitive towards NO and NH_3_, but need dopants or modifications to sense other gases. Graphene, transition metal chalcogenides, boron nitride, transition metal carbides/nitrides, metal organic frameworks, and metal oxide nanosheets as 2D materials represent gas-sensing materials of the future, especially in medical devices, such as breath sensing. This overview covers the most used semiconducting materials in gas sensing, their synthesis methods and morphology, especially oxide nanostructures, heterostructures, and 2D materials, as well as sensor technology and design, application in advance electronic circuits and systems, and research challenges from the perspective of emerging technologies.

## 1. Introduction

The air we breathe is composed of roughly 78% nitrogen (N_2_), 21% oxygen (O_2_), 0.9% argon (Ar), 0.03% carbon dioxide (CO_2_), and 0.07% other gases. Six pollutants have been identified as the main sources of air pollution [1]. In addition to lead and particulate matter, they include carbon monoxide (CO), nitrogen oxides (NO_x_), ozone, and sulfur dioxide (SO_2_). Carbon monoxide is an odorless and colorless gas released during burning. Outdoors it contributes to air pollution, while inside inhalation of large amounts is very dangerous for human health causing dizziness, confusion, unconsciousness, and ultimately death. The fuel burning in power plants and transport vehicles produces nitrogen dioxide (NO_2_). It affects both human health and the environment, causing asthma and acid rain. Sulfur dioxide is mainly the product of fossil fuel burning. The greenhouse effect (heating of the atmosphere) is caused by emission of high levels of greenhouse gases. According to the 2018 Inventory of U.S. Greenhouse emissions and sinks: 1990–2018 carbon dioxide (CO_2_) comprises 81%, methane (CH_4_) 10%, nitrous oxide (N_2_O) 7%, and fluorinated gases 3% of the greenhouse gases causing heating of the atmosphere (the greenhouse effect) [2]. Large amounts of carbon dioxide are emitted into the atmosphere through burning of fossil fuel, solid waste, and chemical reactions such as cement production. Methane is emitted during the production of coal, natural gas, and ores, from municipal solid waste landfills and livestock farms. Hazardous gases include acutely toxic, flammable, oxidizing, corrosive, pyropheric, and dangerously reactive gases. Acutely toxic are carbon monoxide, chlorine, nitrogen dioxide, and phosgene. Flammable gases include fuel flammable hydrogen, acetylene, ammonia, propane, propylene, and methane.

Gas sensors are widely used to detect low concentrations of flammable, explosive, or toxic gases and monitor environmental pollution [3]. Monitoring of gases, humidity, and moisture in the environment surrounding us, medicine, industrial processing, and agriculture is also very important [4]. One illustrative example of the application of gas sensors is sensing hydrogen (H_2_). It is the lightest of all elements and, at standard pressures and temperatures, colorless, odorless, and tasteless. It cannot be detected by human senses. Moreover, it is a highly combustible and flammable gas. With air, hydrogen forms an explosive mixture, which may be ignited in a spontaneous reaction. Hydrogen is applied as fuel and propellant in hydrogen powered vehicles and aerospace operations. Indirectly, hydrogen observation is important in a plethora of spheres ranging from everyday ones like detection of environmental pollution or indication of certain diseases up to an early sign of fire or reactor safety inside nuclear power plants. Hydrogen presence detection and concentration quantification is needed in semiconductor manufacturing and revealing of impending transformer failure in electric power plants. Very different, but equally illustrative examples are the so-called volatile organic compounds (VOCs), which are high vapor pressure organic chemicals. VOCs are both numerous and ubiquitous, and while some of them can cause harm to human health, others present a danger to the environment. Typically, harmful VOCs are not acutely toxic, but if generated inside houses may cause the “sick building” syndrome. Some of them can have compounding long-term health effects and are even carcinogenic. On the other hand, VOCs can also be found inside the human body. Particularly, a breath contains thousands of VOCs, which can originate from either within the body (endogenous VOCs) or from external sources such as diet, prescription drugs, or environmental exposure (exogenous VOCs).

**Figure 1:**
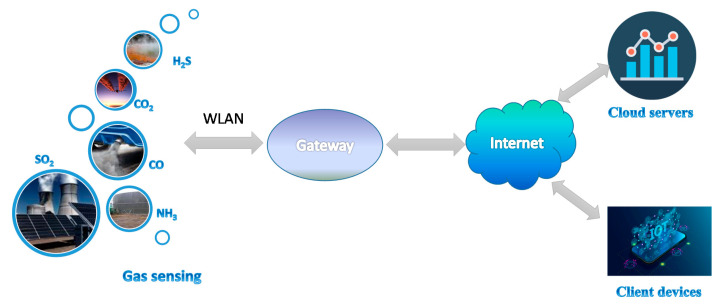
Gas sensing in an internet of things (IoT) network.

Gas sensors and sensor nodes are vital components of advanced communication technologies like the internet of things (IoT), cloud computing, etc. The IoT is a multi-layer technology connecting diverse hardware (smart appliances, smart gadgets, wearable and mobile consumer devices) by middleware to the cloud of thing (CoT) [5] (Figure 1).

The IoT platform (middleware) is flexible “glue” that combines platforms and manages all the interconnections between users, devices (in this case, gas sensor nodes), and applications [6]. Sensor nodes are usually connected in a local area network (LAN), which is the basic/first tier in IoT networking [7]. They are most often equipped by a proprietary or custom wireless local area network (WLAN) transceiver, which connects a node with the gateway [8,9]. It provides the internet access to cloud servers for data processing/storage and client devices using fixed and mobile broadband wireless communication networks (WiMAX, LTE, 5G, etc.) [10].

**Figure 2:**
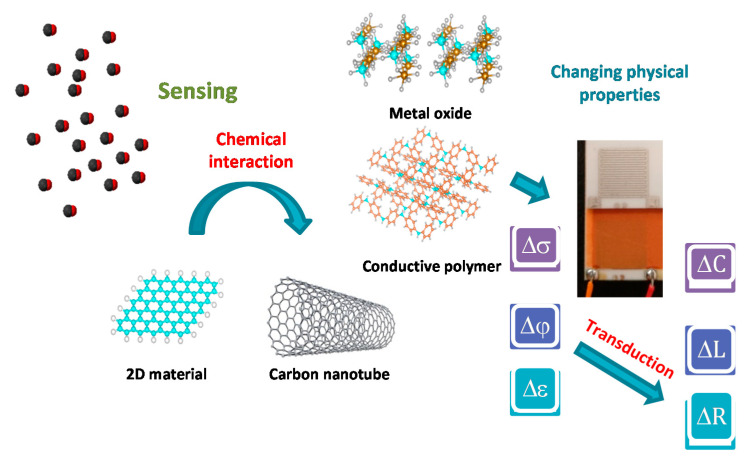
Detection method of semiconductor gas sensing materials.

High sensitivity, fast response/recovery, and good selectivity are generally required of a good sensing material. Development of low cost and reliable sensing devices for the detection of gases, especially at room temperature remains a significant scientific and technological challenge. [11]. Gas sensors based on different sensing materials and methods can be classified according to the detection method. Variation in electric properties is one class of methods [12,13], while other sensing methods include optic, acoustic, chromatographic, and calorimetric gas sensors. When the surface of a semiconductor (metal oxide, carbon nanotube, conducting polymer, 2D material) gas sensor is exposed to the environment, gas interacts with the sensing material and changes its main physical parameters such as conductivity, permittivity, and work function, as shown in Figure 2.

The transducer, another element of the gas sensor, converts these physical parameters into electrical parameters such as resistance, capacitance, and inductance. It produces a sensing voltage or current signal, which magnitude, frequency, and phase can be measured [14].

Thus, in electrically transduced chemical sensors (such as semiconductor gas sensors) where the gas molecules directly interact with the sensing material these interfaces play a key role in defining the sensitivity, stability, and even biocompatibility of sensing devices [15]. The sensing material needs to be designed in such a way that it should have a large exposed surface available for interaction with gas molecules, suitable active sites for binding these molecules and the ability to effectively transduce these binding events into detectable signals. It is also highly beneficial when the mentioned physical effects result in relatively high conductance variability, further implying a higher sensor dynamic range. The sensing material also has to be of good mechanical properties for easy processing.

Semiconducting gas sensors were first used for single gas applications, having advantages that include tunable sensitivity and ability to determine the concentrations of specific constituents, including more recently low energy consumption. However, limitations in the form of high cross sensitivity and poor selectivity to some gases have a tendency to outweigh the advantages. Low precision and selectivity can be overcome by using different sensing materials and implementing multisensor arrays [16,17]. In an electronic nose system, the multiple sensor array component gives an electronic fingerprint response to a particular odor [18,19]. Combined with a pattern recognition module (information processing unit, pattern recognition software, and digital library database) it can recognize odors and discriminate between them. The main task is comparison of the distinct digital response pattern to an odor from a digital reference library database in order to identify and classify it, rather than quantify.

Recent research has focused on the multivariable response principle [17]. The basic concept of this principle is to design a sensor material with a multiple response mechanism to different gases, a multivariable transducer, and data analytics. The multivariable transducer is able to recognize different gas responses and provide independent outputs. Data analytics aims to provide (by multivariate analysis) multicomponent quantification, real with interference and drift. The most commonly implemented multivariate analysis methods include principal component analysis (PCA), discrimination analysis (DA), and artificial neural networks (ANN).

The requirements of modern sensors for IoT can be grouped concerning [17]: (1) reliability; (2) energy consumption; (3) cost; (4) communication capability; and (5) data security. Modern sensors have to be of low cost, so they can be massively applied, be reliable in the sense that they provide accurate readings in diverse environmental conditions, and consume less energy in order to extend battery life. They also have to provide real time communication capability and implement data security mechanisms.

In the following sections, we present an overview of the semiconductor gas-sensing materials (Section 2), a brief description of the gas-sensor fabrication techniques (Section 3) and sensor devices including electronic circuitry and interfaces (Section 4), and a wide range of gas-sensing applications, showcasing where semiconductor gas-sensors are commercially applied and where they are the subject of research for future applications (Section 5).

## 2. Semiconductor Sensing Materials

### 2.1. A Brief History and Current Research Interest

**Figure 3:**
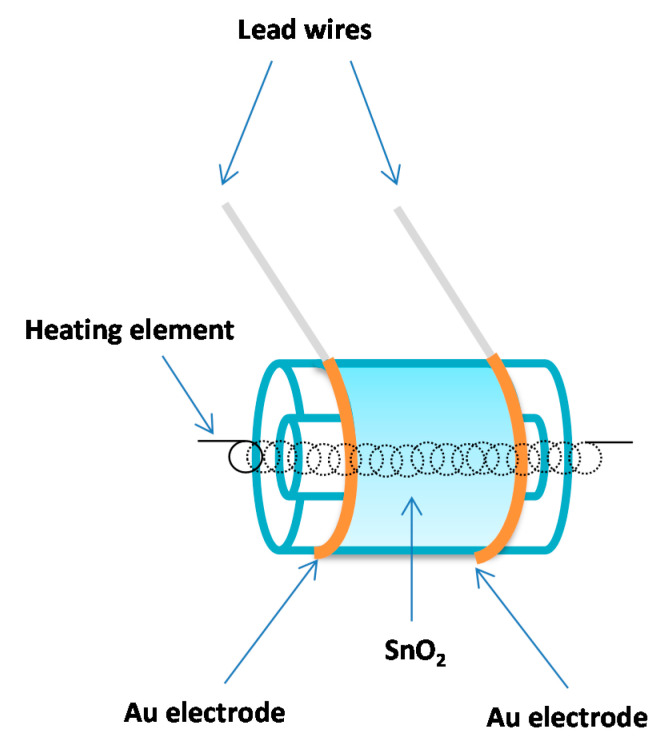
Scheme of Taguchi sensor.

Semiconducting oxides, also better known as metal oxides, are the most commonly used sensing material and still remain so [20]. Different oxide materials have been used and described in literature. Brattain and Bardeen [21] were the first to report on gas sensitive effects on germanium in 1952 making it the first semiconductor used as a gas sensitive material. In 1954, Heiland [22] noted that change in partial pressure of oxygen (or other gases in the atmosphere) had an influence of the semiconducting properties of zinc oxide. In 1962, Seiyama et al. [23] noted on zinc oxide thin films the phenomenon that adsorption or desorption of a gas on the surface of an oxide material changes its conductivity. In 1967, Shaver [24] first described the effects achieved by doping tungsten oxide thin films with noble metals, primarily platinum. In 1971, Taguchi [25] applied tin-oxide in the first commercial sensor (Figure 3).

Figaro Inc. commercialized these devices as gas sensors monitoring the presence of hazardous levels of explosive gases in homes [20]. They were widely used to prevent fires. This resulted in a widespread application of semiconductor gas sensors. Over time commercially produced metal oxide gas sensors have reduced in size being produced in different technologies, starting with tube-type sensors in the eighties of the last century, through screen-printed sensors produced in the nineties and completing with state-of-the art micro-electro-mechanical system (MEMS) sensors produced today. One of the big producers of metal oxide gas sensors, Figaro Engineering Inc., still sells some of the sensor designs produced in the 1980s. Application of advanced fabrication technologies has enabled sensor miniaturization and development of sensor arrays with enhanced selectivity and sensitivity [26].

**Figure 4:**
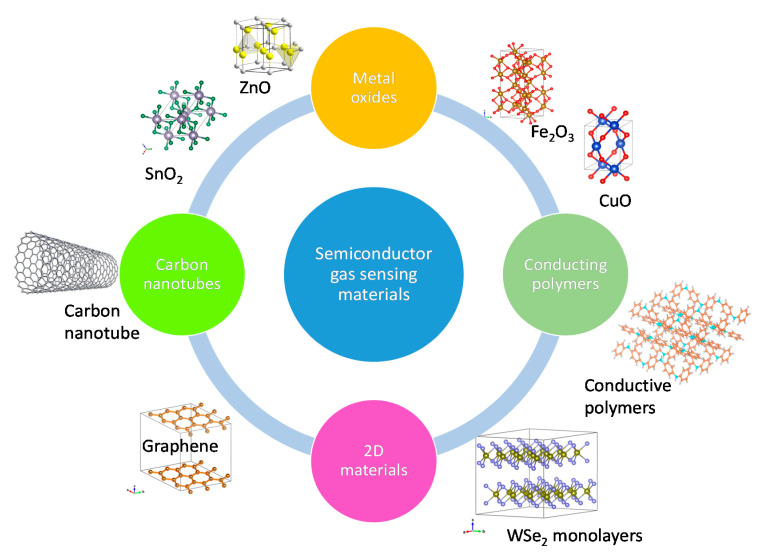
Semiconducting gas sensing materials.

Since the 1980s, research into different gas sensing materials has been quite intense and widespread. It has diversified from metal oxides that are still the subject of much research especially nanostructures [11,14], to conducting polymers [18,27], since 2000 to carbon nanotubes [28,29] and continued with their composites [30,31,32], and more recently 2D materials [15,33], as shown in Figure 4. The crystalline structures were drawn using VESTA [34]. These novel materials are still in the research phase rather than applied widely in commercial applications. In addition to promising properties, they have many shortcomings that need to be dealt with first. Application of conducting polymers is hampered by the fact that fabrication is complicated and time consuming [18]. Due to oxidation, the lifetime of gas sensing devices based on conducting polymers is relatively short (9–18 months) compared to metal oxide gas sensors. In the case of 2D materials fabrication costs are also high and other problems are detection limits, poor selectivity, and slow recovery of the gas sensor baseline [35].

### 2.2. Metal Oxide Semiconductors

An overview of the investigated metal oxide semiconductors shows that over the years, n-type semiconducting oxides [18] like tin oxide (SnO_2_) [36,37], zinc oxide (ZnO) [38,39], anatase (TiO_2_) [40,41], hematite (α-Fe_2_O_3_) [11], and tungsten oxide (WO_3_) [42,43] and, to a lesser degree, p-type semiconducting oxides such as CuO [44,45], NiO [46,47], and Cr_2_O_3_ [48] or Co_3_O_4_ [49] have been extensively studied. Complex (mixed metal) oxides have also been investigated [50] such as perovskites [51,52] and cubic spinels such as ferrites [11,53,54] and stannates [52]. Much research has focused on improving the sensing properties of metal oxides, with doping being one of the paths, some examples are Sr-doped Fe_2_O_3_ [55,56], Sm-doped CoFe_2_O_4_ [57], and Nb-doped TiO_2_ [58].

Advantages of using metal oxide semiconductors as gas sensing materials include low cost, easy fabrication, simplicity of use, ability to detect different gases including flammable and toxic gases. Disadvantages or limitations of these sensing materials include poor selectivity and cross-selectivity, low sensitivity to lower gas concentrations in other words detection limit, high power consumption, baseline resistance drift, and high operating temperature [11].

Simple techniques such as solid-state synthesis or high-energy ball milling are still applied for synthesis of metal oxides for gas sensing [59,60]. Solution-based synthesis methods such as hydrothermal and solvothermal synthesis [37,39,45], sol-gel synthesis [41,61,62], or electrospinning [63] are the subject of much research as they can create a diverse spectrum of nanostructures and heterostructures [64]. In the form of thin films, metal oxides for gas sensing are commonly synthesized using thermal evaporation [65], sputtering [66], and chemical vapor deposition [36] techniques.

#### 2.2.1. Tin Oxide (SnO_2_)

**Figure 5:**
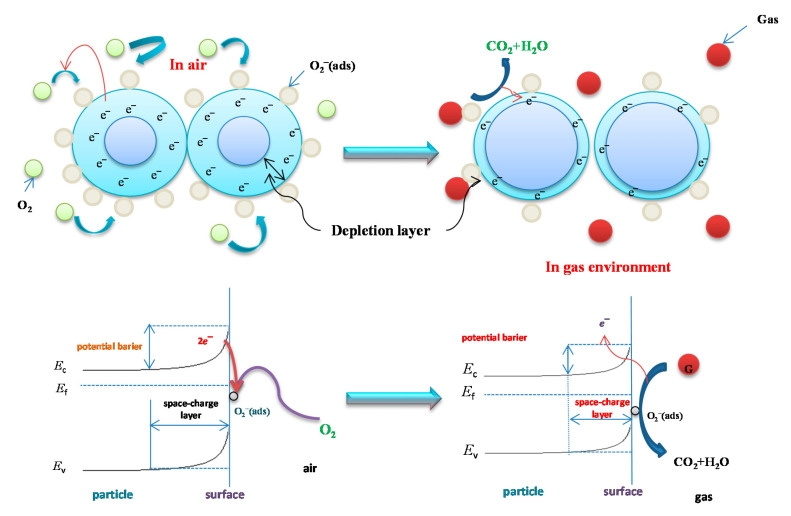
Schematic diagram of the sensing mechanism of n-type semiconducting metal oxide nanostructures for reducing gas.

Tin oxide (SnO_2_) is the most extensively studied metal oxide gas sensing semiconductor. It is widely applied in practical commercial gas sensor devices. Tin oxide is a wide-band gap (3.6 eV) semiconductor. It is morphologically and chemically stable. Electrons are the majority charge carriers in n-type semiconductors such as tin-oxide (Figure 5).

Interaction with a reducing gas gives rise to an increase in conductivity, while an oxidizing gas serves to deplete the sensing layer of charge carrying electrons, resulting in a decrease in conductivity [67]. Surface states in SnO_2_ behave as electron donors or acceptors. Electron donors or acceptors at the surface cause an exchange of electrons within the interior of the semiconductor forming a space charge layer close to the surface. Gas interacts better with a porous surface, so this is a prerequisite of a good sensing material [68,69]. The specific surface of a gas sensing material needs to be high to achieve increased reaction with the target gas depending on the material grain size. Due to its high sensitivity to different gases, tin-oxide-based sensors can detect low levels of gas concentration, though tin-oxides suffer from low selectivity.

Significant research has focused on doping SnO_2_ with noble metals, such as Pt, Pd, and Au or other metal ions, for instance Ni, Fe, and Cu. Introduction of dopants, such as Pt and Pd, evenly dispersed on SnO_2_ grain surfaces, has led to improved gas sensing performance of SnO_2_ to gases such as CO, CH_4_, and NO_2_ [70]. Pt doping of SnO_2_ has resulted in lower overall resistance, increased sensitivity, and faster response to ethanol [71]. Pt doping of SnO_2_ nanofibers obtained by electrospinning improved significantly the response to H_2_S [72]. Pd doping of SnO_2_ nanorod thin films obtained by chemical vapor deposition advanced by plasma improved gas sensing to H_2_ at 300 °C [73]. The Pd doping concentration and sensor operating temperature had a decisive influence on the sensitivity of Pd-doped SnO_2_ hollow nanofibers obtained by electrospinning to H_2_, CO, CH_4_, and C_2_H_5_OH [74]. Similarly, the operating temperature decreased and sensor response to H_2_ increased with increasing Pd dopant concentration of Pd-doped SnO_2_ nanowires [75]. Doping SnO_2_ thick and thin films with Au, lowered the sensor operating temperature and improved sensitivity and selectivity to CO [76,77]. Doping with Au changed the morphology of SnO_2_ thin films not only reducing grain size, but resulting in a mesoporous structure with clearly separated grains, enhancing sensitivity and selectivity to reducing gases, such as CO [77]. A higher surface area and reduced grain size was achieved by doping SnO_2_ with Fe or low concentrations of Ni (2–4 mol.%) showing improved response to ethanol [78], or n-butanol and formaldehyde [79], respectively.

Performance enhancement of tin-oxide-based sensors has also been achieved by tuning the synthesis process [36,80], synthesizing hierarchical SnO_2_ nanofiber/nanosheets [81], and tubular nanomembranes [82]. Nanocomposites of multi or single walled carbon nanotubes with SnO_2_ achieved room temperature detection of NO_2_ [30,83]. Nanocomposites/heterostructures of SnO_2_/MOx (M = Zn, Ga, and W) in the form of electrospun nanotubes and nanofibers assembled in a gas sensor array have shown high sensitivity and selectivity to ethanol, acetone, and xylene using the method of matrix manipulation [64].

Ongoing research has shown that doping SnO_2_ nanocomposites with noble metals or metal ions remains a way for enhancing gas sensing performance. Fast, sensitive, and selective detection of acetone was achieved by a nanocomposite of Ni-doped SnO_2_ and graphene at 350 °C [84]. Cu doping of SnO_2_ nanograins led to tailoring of surface defects and improvement of the surface potential barrier [85]. When these nanograins were enveloped with polypyrrole in an organic/inorganic nanohybrid, room temperature sensitivity to H_2_S was achieved [86].

#### 2.2.2. Zinc Oxide (ZnO)

Nanoscience has brought many innovations to material processing offering controllable manipulation of materials at the molecular level [18]. Nanostructures offer a smaller particle size that leads to a larger surface are available for interaction with the target gas. Then the resulting sensors can be smaller, have a lower weight, lower power requirement, and greater sensitivity. With a direct band gap of 3.37 eV and high exciton binding energy responsible for efficient ultraviolet emission zinc oxide (ZnO) has been extensively investigated for a wide range of applications, including gas sensing. Even though the first results on ZnO gas sensing capabilities were obtained in 1962 by Seiyama in 1962 [23], with the advent of modern nanotechnology, zinc oxide has been rediscovered as a sensor material, becoming again the focus of much research. It can be synthesized using different methods into a variety of nanostructure morphologies such as nanoparticles (traditional rounded 3D shape), nanorods [87,88] nanowires, nanobelts, nanoterapods [36], nanoflowers [39], nanowhiskers [89], and many others. Recent research has focused on 1D ZnO nanorods and nanowires grown by a vapor–liquid–solid method using Au, Pt, Ag, and Cu nanoparticles as catalysts [90]. These morphologies have improved surface chemical characteristics, ascribed to size confinement in two dimensions, resulting in a better crystallinity and larger surface to volume ratio [36]. Surface modification, doping, and light activation of synthesized ZnO nanostructures can lead to room temperature gas sensing that is significant when sensing flammable and explosive gases [91].

#### 2.2.3. Copper Oxide (CuO)

Copper oxide (CuO), a p-type semiconductor, has been investigated for a wide range of applications including gas sensing. In p-type semiconductors the majority charge carriers are holes, thus high gas response is not achieved in the same way as for n-type semiconductors. In this case, the key morphological parameter for attaining high gas sensitivity is high inter-particle connectivity [49]. CuO and other p-type semiconductor metal oxides such as NiO, Cr_2_O_3_, or Mn_3_O_4_ are able to catalyze the oxidation of volatile organic compounds resulting in high selectivity to these gases. Another advantage of p-type sensors is their tolerance to humidity. Different CuO morphologies have been obtained using different synthesis methods, where urchin-like, fiber-like, and nanorod were obtained using the microwave method, different bases, and solvents [44], while application of the hydrothermal method gave CuO nanoparticles sensitive to methanol, ethanol, and acetone [45].

#### 2.2.4. Nickel Oxide (NiO)

Nickel oxide (NiO), also a p-type semiconductor with a wide band gap (3.6–4.2 eV) has been the focus of recent research in the form of 0–3D nanostructures or heterostructures as a gas sensing material, especially for hazardous and toxic gases [92]. NiO thin films are able to operate at high temperatures (>500 °C) and detect H_2_ [47]. A high response ratio to ammonia (NH_3_), fast response and recovery time in the temperature interval 250–350 °C was obtained for NiO thin films fabricated by RF magnetron sputtering [93]. In the form of nanowires with a high aspect ratio NiO has shown promising gas sensing performance towards toluene, ethanol, acetone, methanol and triethylamine at 350 °C [94], while nanowires grown using Pt, Pl, and Au as growth catalysts have shown good sensing properties towards hydrogen (H_2_) and CO at 300 °C and acetone at 500 °C [95]. Hydrothermal synthesis was used to obtain a multiple continuous networking nanofiber structure with enhanced gas sensing properties [46], confirming the influence of morphology and inter-particle connectivity in p-type semiconducting gas sensing materials. Gas sensing properties of NiO have been improved by doping, such as Al-NiO nanorod flowers obtained by solvothermal synthesis [96]. Doping with Al increased the number of active oxygen sites improving adsorption towards ethanol.

#### 2.2.5. Chromium Oxide (Cr_2_O_3_)

Chromium oxide (Cr_2_O_3_), also a p-type wide band gap semiconductor (3.4 eV) has been investigated for gas sensing [48]. Recent research has focused on enhanced sensing of toluene (a neurotoxic volatile organic compound—VOC) by Cr_2_O_3_ microspheres [97]. Addition of noble metal catalysts in the form of nanoparticles decorating the surface of Cr_2_O_3_ has for example resulted in lowering the optimal operating temperature for sensing triethylamine to 100 °C for Ag decorated Cr_2_O_3_ microspheres [98], while enhanced sensitivity and selectivity was accomplished for yolk shell spheres decorated with Au nanoparticles [99]. In composites, such as Cr_2_O_3_-WO_3_, the presence of chromium ions improved gas selectivity to NO_2_ in NO_x_ mixtures [100]. Highly sensitive p-type gas sensors can be obtained by electrospining Cr_2_O_3_-Co_3_O_4_ nanofibers achieving high inter-particle contact areas [49].

#### 2.2.6. Zinc stannate (ZnSnO_3_ and Zn_2_SnO_4_)

Zinc stannate (zinc tin oxide) is a ternary (mixed metal) oxide characterized by a high electron mobility, high electron conductivity, good stability, and attractive optical properties [52]. It has two polymorphs. The crystal structure of the first polymorph (ZnSnO_3_) can be of the lithium niobhate type or of the cubic perovskite type, while the second polymorph crystallizes in a simpler cubic inverse spinel structure (Zn_2_SnO_4_). Both polymorphs can be applied as a gas sensing material, especially for reducing and combustible gases, and also thriethylamine, ethanol formaldehyde and others. Zinc stannate in both polymorph forms has been synthesized in a wide variety of nanostructures such as 1D nanowires, 2D nanoplatelets and nanosheets, 3D polyhedral and hierarchical architectures suitable for gas sensing applications.

#### 2.2.7. Heterostructures

A relatively new path for achieving improved properties of gas sensing materials in terms of sensitivity and selectivity has been to combine metal oxides together into heterostructures [101]. The simplest heterostructure form is a mixed compound representing a mixture of two metal oxides without a specific distribution. Many such heterostructures have been investigated including TiO_2_-Fe_2_O_3_ [102], WO_3_-TiO_2_ [103], TiO_2_-SnO_2_ [104], ZnO-SnO_2_ [105], Zn_2_SnO_4_-SnO_2_ [106], MgFe_2_O_4_-Fe_2_O_3_-SnO_2_ [107], and many others. Self-assembly of Fe^3+^ ions on the surface of Cu@carbon microspheres, when transformed by thermal oxidation and a solid-state reaction form a CuFe_2_O_4_-α-Fe_2_O_3_ composite sensitive to low amounts of acetone [108]. Limited sensor response was achieved by stacked heterostructures based on bi- or multi-layered films.

Heterostructures containing a p-type and n-type semiconducting metal oxide result in not only mixing different physical and chemical properties of the two component metal oxides, but in the formation of p-n (or n-p) heterojunctions due to the difference in Fermi levels causing band bending and formation of a potential energy barrier at interfaces [109]. This barrier changes when the composite material is exposed to oxidizing or reducing gases and has brought about noticeable improvement in gas sensing performance. One such composite is NiO-SnO_2_. In the form of 3D microflowers, with the added benefit from the catalytic effect of NiO, enhanced sensitivity to formaldehyde was achieved at 100 °C combined with fast response and recovery, good reproducibility and stability [110]. Improved sensitivity to NO_2_ and C_6_H_6_ was achieved by nanowebs, with the composition 0.5NiO-0.5SnO_2_ [109].

Decoration of a host material with nanoparticles of a secondary material is another type of heterostructure [111]. Core-shell structures aim to cover the host material with a secondary phase in order to maximize the interfacial area [112]. Research of these structures has included WO_3_-SnO_2_, TiO_2_-SnO_2_ core-shell nanofibers [113,114] and α-Fe_2_O_3_/TiO_2_ core-shell microspheres [115]. Electrospun SnO_2_/MO_x_ heterostructures have been synthesized, characterized, and integrated in highly sensitive gas sensor arrays [64]. Heterostructured nanotubes obtained by electrospinning NiO (p-type) and ZnO (n-type) metal oxides have shown shorter response and recovery times and increased sensor response to H_2_S [116].

### 2.3. Conducting Polymers

Conducting polymers (also known as intrinsic conducting polymers) such as polyaniline (PANI), polypyrrole (PPy), and poly(3,4-ethylene edioxythiopene) have been investigated as sensing materials and used as active layers for gas sensing since the 1980s [27]. Conducting polymers as gas sensing materials offer many advantages such as room temperature operation, good mechanical properties, and easy synthesis. Their main disadvantage for a wide practical application is long-term instability and irreversibility and low selectivity. Over time, the sensor response decreases and this process is not reversible, thus the lifetime of sensors that use conducting polymers is not long. Their stability is greatly influenced by ambient conditions such as change in humidity or temperature that can influence chemical and physical properties of the gas sensing conducting polymer layer, also interaction between the analyte and conducting polymer can influence stability. These interactions can cause a swelling effect in the conducting polymer layers that has an impact on the electrical resistance and sensing ability.

Conducting polymers have been widely investigated for the development of sensor arrays [14,18], such as the proposed electronic nose composed of polypyrrole gas sensors for detecting and discriminating between methanol, ethanol, acetone, 2-butanone, and 2-pentanone [117] or methanol, acetone, chloroform, benzene, and ethanol [118]. Conductive polymers are sensitive to redox-active gases such as NH_3_, NO_2_, and some organic vapors, though they have difficulty to detect volatile organic compounds (VOC). Highly sensitive (10 ppm NO_2_) gas sensors using polypyrrole films were proposed by Navale et al. [119], while Su et al. [120] designed a flexible polypyrrole sensor for NH_3_ assembled in situ on a modified PET substrate. One of the advantages of conducting polymers are tunable properties. They can be improved using chemical functionalization and nanostructuring [121]. Conductivity of conducting polymers can be further improved by doping or the formation of composites [122]. Conducting polymer composites can be a composite of two polymers (conducting and insulating) such as polypyrrole and poly(vinyl alcohol) obtained by electrochemical polymerization showing a noticeable change in resistance when exposed to NH_3_ [123].

Carbon black can also be dispersed in an insulating polymer matrix resulting in a conducting polymer composite [18]. They are a commercially available family of sensors and can be produced with high humidity tolerance and due to the varied choice of insulating polymers with a broad range of selectivity. They are often applied as an array in an electronic nose, such as the Jet Propulsion Laboratory’s electronic nose (JPL ENose) used for air-quality monitoring (20 analytes) in space stations [124,125], or in the form of a portable nose system [126].

Composites with metal oxides, especially nanoparticles such as SnO_2_ and/or TiO_2_, have resulted in conducting polymer composites for CO gas detection [127], while thin film camphor sulfonic acid doped polyaniline-ZnO nanocomposites obtained using spin coating were able to detect NH_3_ at room temperature [31]. Other functional materials have been also combined into composites with conducting polymers, such as carbon nanotubes [32] or graphene [128]. Flexible multisensory platforms utilizing conducting polymer nanocomposite sensors can be designed, manufactured, and integrated at a relatively low cost onto smart textiles or radiofrequency identification (RFID) tags for logistic applications.

### 2.4. Carbon Nanotubes

Studies on applying carbon nanotubes (CNTs) as gas sensing materials started twenty years ago [129,130]. Representing seamless cylinders formed by wrapping of graphene sheets along the axial direction, they can be single-walled (SWCNTs) formed of one sheet when all atoms behave as surface atoms, or multi-walled (MWCNTs) formed of several sheets where only atoms in the outermost layer are responsible for the sensor response [131]. Carbon nanotubes have good chemical and mechanical stability, electronic properties suitable for gas sensing applications coupled with a high surface to volume ratio [14]. As such, they are marked as next generation gas sensors that can make a difference in the gas sensing market [29]. CNTs are sensitive towards strong electron withdrawing or donating gases such as NH_3_ and NO_2_. There are technological barriers that hinder their commercialization. Their synthesis is costly and still challenging as it is difficult to grow continuously defect free nanotubes [28]. Another issue is interaction of CNT surface with oxygen/water molecules that can have an influence on the sensor response to target gases. One method to counter this problem is ultraviolet (UV) light illumination [29]. The change in electrical properties of CNTs when exposed to gas molecules is attributed to charge transfer between the nanotubes and molecules (acting as electron donors or acceptors). The preparation process and technique have a significant influence on sensor behavior and properties, so variations can be crucial for the stability of devices based on carbon nanotubes. Another disadvantage of CNT gas sensors is a slow response and recovery due to the nature of the processes of gas adsorption and desorption by this material [28]. In addition to research on gas sensors using SWCNTs and MWCNTs, for example MWCNTs for hydrogen detection [132] research has focused on incorporating CNTs into various matrix materials. These include polymers, for example, multi-walled carbon nanotubes (MWCNTs) reinforced with PEDOT and PANI that achieved good selectivity to ammonia, but recovery was still poor [32], metal oxides, such as MWCNT/SnO_2_ composites for detecting hydrogen [30], or NO_2_ at room temperature [133]. Decoration of CNTs with noble metal nanoparticles has led to improved gas sensing response to some gas molecules, such as the enhanced response to H_2_ achieved by SWCNTs decorated with Pd [134].

### 2.5. 2D Materials

Graphene is a 2D monolayer composed of bonded carbon atoms with high electron mobility at room temperature [135]. The 2D structure of graphene makes every carbon atom a surface atom resulting in the fact that electron transport through graphene is very sensitive to adsorbed molecules. In 2007, graphene was marked as a promising gas sensing material with exceptional electrical and mechanical properties [136]. Compared to CNTs, its sheet like structure is more suitable for depositing and stabilizing nanoparticles (metal or metal oxide) and can be easily integrated into sensor devices [18]. Interest in graphene as a 2D material for gas sensing has opened the door to a whole series of 2D analogues that include transitional metal chalcogenides (TMDs), hexagonal boron nitride (hBN), black phosphorous (BP), and transition metal carbides/nitrides (MXene family of materials) all with gas sensing potential [14]. The single layer architecture of these 2D structures enables full contact of gas molecules with their surface.

TMDs are a group of materials that show electronic properties similar to graphene, but have a bandgap value more suitable for practical device applications than graphene [137]. Disulfides have been investigated the most, and have shown potentially excellent gas sensing properties in terms of high sensitivity, fast response and good stability. An example is MoS_2_ synthesized in the form of hierarchical microspheres forming a layered structure and large surface area showed good gas sensing ability to hydrogen at a lower operating temperature [138]. MXenes have been the focus of much recent research as a gas sensing material [139]. Recent research has shown that vanadium carbide MXene flakes (V_2_CT_x_) can be applied for detection of very low amounts of methane and hydrogen at room temperature [140].

The future for gas sensing materials lies in diverse types of heterostructures probably involving combinations of the above-described sensing materials. One path is to combine several types of 2D materials, for example graphene with another 2D inorganic material, such as an MXene. Another example is synthesized hybrid fibers of Ti_3_C_2_T_x_/graphene that have shown exceptional electrical and mechanical properties combined with room temperature gas sensing of NH_3_ suitable for application in flexible wearable electronics [141].

Metal organic frameworks (MOFs) are porous functional materials composed of metal ions with strong bonds and organic ligands [142]. Pyrolysis or calcination is used to convert these precursor structures into diverse metal oxide sensor nanostructures and nanocomposites [143]. These materials could offer a large pore volume and surface area and good chemical stability. An example is ZnFe_2_O_4_ nanorods obtained from a precursor Zn/Fe metal organic framework as a precursor. Combined with reduced graphene oxide, a p-n heterostructure was obtained showing ability to detect SO_2_ at room temperature [144].

Research in terms of improving the gas sensing material, design and technology could also be in advanced nanofabrication technologies and geometrically structured nanomaterials [145].

## 3. Sensor Fabrication

Printing is one of the methods used commercially to fabricate semiconducting oxide sensing layers, but also other components of the electronic sensing device [14]. Similar to graphic printing, printing of semiconductor oxide sensing layers entails incorporation of semiconducting oxides into ink systems and yields high-quality electron devices that are thin, lightweight, flexible, low-cost, and environment friendly.

Screen-printing is a contact printing method commonly used to fabricate a thick film-sensing layer over an insulating substrate with electrodes and a heater to fabricate a gas-sensing element. This method involves pushing an ink through a porous layer or mesh masked by a resin to produce the required layout on the substrate. It is usually a thick film layer of the sensing materials in the form of an ink or paste made as a mixture of the oxide sensing material and organic vehicles (solvent, additives and binder). The organic vehicles in the paste usually need to be burnt out after the printing process to leave only the oxide material. Screen-printing can be flat bed or rotary and made semi or fully automated to increase printing speed. This versatile technique is used in printed electronics on a variety of substrates, including flexible [146]. This technique can be used to print low resistance semiconducting materials, such as conducting polymers.

In non-contact printing methods, the sensing material is directly deposited on a substrate using a nozzle or print head. With the print head and substrate both in motion, a desired pattern can be created without the need for a mask or template [14]. Inkjet printing consists of achieving a pattern of a desired image by ejecting ink drops form an ink reservoir onto a substrate. It can be continuous or drop on demand. Both methods are slower than screen-printing, though ink-jet printing has been used to fabricate large-area sensors as a wearable electronic e-skin [147]. Aerosol jet printing deposits an aerosol to make the desired pattern through a print-head nozzle and is often used for printing electrodes. There are also various types of 3D printing.

A lot of research has gone into making and formulating inks for printing to be suitable for printing different designs on different substrates. Inks for printing oxide sensor layers can be made in two different ways. One way is to use previously synthesized or purchased powder and disperse it into a solvent. Necessary additives and a binder can also be added to the solvent. The second way is to use metal precursors in the solvent and make the ink, print it and then synthesize the metal oxide by thermal treatment/calcination of the printed design. Both have been the subject of much research [148,149,150].

**Figure 6:**
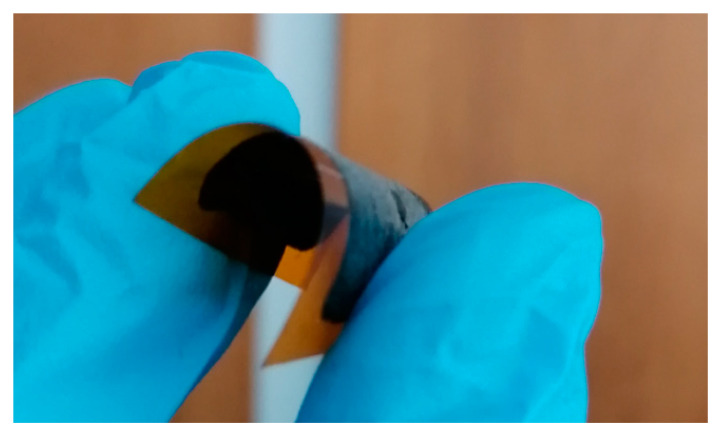
Example of the semiconducting oxide thick film on Kapton substrate.

With the development of flexible electronics and flexible gas sensors on plastic substrates such as polyimide, Kapton (Figure 6), polyethylene (PET) thermal treatment for binder removal needs to be at temperatures often below 100 °C, i.e., compatible with the temperature the plastic supports [151]. This entails careful selection of the binder. Flexible sensors based on metal oxide nanostructures show high sensitivity, low production cost, ease of fabrication, and eco-friendliness [152,153]. They are used in wearable electronics.

Transfer printing is a method of printing the sensing material on a different material, often a polymeric-based stamp that is then transferred to the sensing device [146]. Hyodo et al. [154] used slide-off transfer printing to stack layers of semiconducting metal oxides. While a single layer of screen printed TiO_2_ showed high resistance and low sensitivity to H_2_ in the temperature interval 200–600 °C, addition of a noble metal (Au, Pt, Pd, Rt) doped SnO_2_ layer improved sensitivity to H_2_ by changing the electron conduction path. The CVD method was used to synthesize graphene on Cu foils and then transferred on to Si wafers, decorated with Pd using electron beam evaporation. High sensitivity, fast response, and recovery for multiple cycles to H_2_ were shown [155]. SnO_2_ nanowire-based sensors with high sensitivity to H_2_S were obtained by transfer printing using a polymer-based stamp (polydimethylsiloxane-PDMS) [156]. SnO_2_ nanowires were fabricated using a two-step method involving spray pyrolysis on the Si substrate. The nanowires were harvested from the Si substrate and transferred to two types of sensor devices (Si and micro hotplate-based) using different manufacturing techniques achieving small difference in response to H_2_S, and a different optimal temperature (300 °C and 350 °C) for the best response to CO gas.

An alternative to transfer printing is direct growth of the sensing material on the sensing device. This method eliminates the need for manual transfer and is simpler as it results in immediate device fabrication, but the procedure of achieving direct growth is often complex too. Shekhurev et al. [157] have proposed a method for simple CVD growth of graphene nanoribbons on high temperature resistant substrates (Si/SiO_2_, silica glass or metals). The fabricated grapheme nanoribbon devices were sensitive to a variety of VOCs. Thermal oxidation of electroplated copper was applied to achieve on-chip growth of CuO nanowires, achieving a high sensitivity to H_2_S and ability to detect low concentrations (10 ppm) of CO gas [158].

## 4. Sensor Device

**Figure 7:**
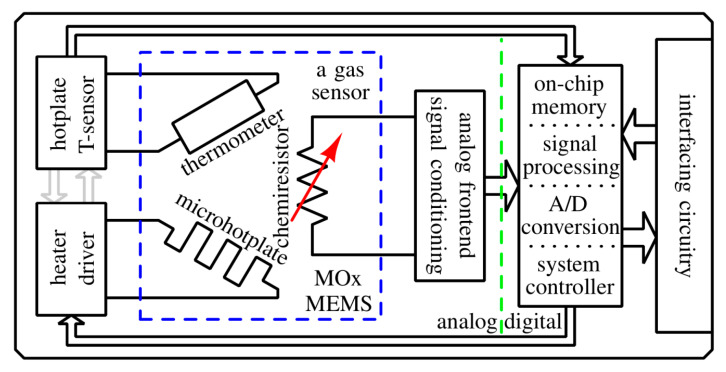
Chemiresistive MOx gas sensor model and interface circuitry.

The sensor performance is greatly influenced by the design of the sensor device and materials. Gas sensors using semiconducting oxide materials need to have a heater as their operating temperature is often in the range from 150–450 °C. In the most common conventional designs, the electrode is printed on one side—the top of the substrate, while the heater is printed on the bottom side of the substrate. Electrodes and heaters are printed using gold, platinum, or palladium-silver paste, while the substrate is conventionally silicon/silicon dioxide or alumina. This type of gas sensor consumes much energy due to heating the thick substrate. This disadvantage can be reduced using complementary metal-oxide-semiconductor (CMOS) compatible MEMS fabrication processes to obtain a thinner substrate supporting the interdigitated electrode structures. The MEMS technology enables low power consumption, high sensitivity, miniaturization, and wafer-based manufacturing. This reduces the production costs. This technology is easily reproducible and scalable. Semiconductor materials are deposited on MEMS substrates in the form of thick and thin films in same way as on silicon or alumina. Screen and inkjet printing or drop casting is usually used for thick film deposition, while sputtering is most often used for creating thin films [42,150]. Surface micromachining has been applied aiming to produce uniform and reliable metal oxide (SnO_2_) gas sensors [159], while nanofabrication technology has been applied to achieve a nanowire heater with ultralow power consumption [160]. Figure 7 shows the model of a chemiresistive MOx gas sensor.

### 4.1. Gas Sensor Signal Conditioning and Interfaces

Design of semiconductor gas sensor interface circuitry depends on implementation approach, which can be a hybrid or monolithic.

#### 4.1.1. Hybrid

Traditionally, integration is realized with the so-called multi-chip approach in which the sensor and circuits are designed and fabricated on separate chips. This two-chip solution has multiple advantages. First, this implementation scenario enables independent adjustment and optimization of the gas sensor and the interface circuitry thus providing much more flexibility in the design and fabrication, leading to shorter development cycles. Additionally, hybrid integration also enables deposition of the gas sensing layer at higher temperatures or with materials, e.g., gold or platinum, and processes that are not compatible with the baseline CMOS silicon technology in that manner providing extra resilience in terms of exploited materials. Furthermore, if there is a problem with the sensing device, the same circuitry can be reused, thus enhancing manufacturing yield. However, extra cost is incurred by the complex packaging. In addition, parasitic capacitances and inductances associated with long bonding wires and interconnect are undesirable and can give rise to increased noise and signal degradation. Moreover, the hybrid approach is less robust and more expensive with respect to single-chip implementation particularly when considering high volume production.

#### 4.1.2. Monolithic

A more recent and a more advanced method is the so-called monolithic approach, which provides design and fabrication of both, the sensor and interface circuitry, on a single substrate. This single-chip solution enhances the gas sensor performance by reducing its overall size, power consumption, and noise. It is also more cost-efficient and hence more commercially attractive in high unit volumes. Additional challenges arise from a prolonged and costly development cycle. In addition, a potential fault in either a sensor or the nearby circuitry will result in the complete chip failure. Thermal isolation is crucial since the main challenge is that the operating temperature of the metal oxide sensor component exceeds the maximum operating temperature of the CMOS-based silicon integrated circuits but, if a proper isolation mechanism is employed [161], only several degrees difference between the sensor circuit and ambient temperatures can be achieved.

### 4.2. Driving, Sensing, and Control Circuitry

As mentioned above, metal oxide gas sensors operate usually in a temperature range of 150–450 °C, incorporating an integrated heater. The gas sensor response greatly depends on achieving a precise temperature, so heater driving and temperature control is a crucial part of the overall gas sensor system. The temperature control circuitry becomes more challenging when taking into account that semiconductor metal oxide sensors can be operated either in an isothermal (that is, constant temperature) or a temperature-modulated regime, the latter being more complex providing insight into a temporal response pattern, which can discriminate different analytes [162].

#### 4.2.1. Heater Driver

Heaters are driven by either a current or a voltage source. Circuits are designed to provide a source either at a static (e.g., DC) or modulated (e.g., AC or pulsed) level. In the case of a voltage drive, commonly a bandgap voltage reference and a current limiter are required to provide an accurate and stable voltage and avoid any overheating damage. In the case of current drive, a current mirror is employed. In both cases, it is desirable to measure the power through the heater and provide an indication of temperature and general operating status [163].

#### 4.2.2. Temperature Sensor

Since the temperature of the sensing material plays a vital role in improving the selectivity of almost any gas sensor, either a dedicated temperature sensor or a temperature dependent heater resistor can be exploited to estimate the actual temperature of the heater itself. Even though separate temperature sensors bring additional circuit complexity and cost, they are often a much more reliable approach especially when tied with the pulsed driving. In principle, the temperature accuracy of a gas sensor directly corresponds to the accuracy of the temperature sensor if the temperature is controlled inside the feedback loop. Nevertheless, the actual surface temperature is not only a function of the power dissipated by the heater, but the ambient conditions too.

#### 4.2.3. Controller

With incorporation of a microheater and temperature sensor of some sort, there are several ways to monitor and control the heater temperature. The heater can be controlled in a binary on-off mode (also referred to as the “bang-bang” controller), proportional mode or proportional integral derivative (PID) mode, which eliminates the steady-state error while offering rapid response time without overshoot. In cases when the temperature sensor readings are digitized and compared to a digital pre-set value, a digital controller can be designed to achieve very accurate and flexible soft-programmable control.

### 4.3. Sensing Material Measurement and Readout Interface

Interfacing circuitry design is one of the most challenging components mainly because it has to: (i) handle the precision and dynamic range of the gas sensing element whose baseline sheet resistance can vary from kΩ/Υ all the way up to GΩ/Υ and (ii) compensate for the drift in the baseline resistance of the sensing material. The simplest scheme involves resistance-to-voltage conversion through either a resistive voltage divider or a Wheatstone bridge. When seeking full integration, these techniques are not ideal, as they require either a resistor bank circuit with a very large value or trimming or variable resistors to cover a wide range and match the sensing material resistance.

Another approach is to use the resistance-to-frequency conversion instead, but in order to cover a very high dynamic range the parasitic capacitance associated with the sensing material has to be retrieved and isolated [164] to avoid resistance measurement contamination in the high frequency portion.

A relatively simple idea is to use a logarithmic converter (utilizing exponential characteristics of a diode) to compress the large dynamic range but it inevitably compromises accuracy.

By rule, contemporary sensors include analogue front-end circuitry, which consists of amplifiers that increase sensor signal fidelity as well as analogue-to-digital (A/D) converters.

Digital sensor platforms are becoming increasingly popular [165] as they effectively co-integrate analogue and digital electronics together with a microhotplate and the sensing elements onto a single die. They allow more advanced signal processing circuitry in the digital domain and incorporate a digital interface that greatly simplifies integration into different applications, since the output signal can be directly used without further processing [166].

## 5. Application

The current global gas sensor market size has been roughly estimated [167,168] to be worth somewhere in-between one and two billion euros and is anticipated to have the annual growth rate of about 5–10% in the forthcoming decade. This effective market doubling in the next ten years is partially going to be driven by regulatory initiatives in developed markets, which will lead to increased demand for smart and wireless gas sensors. The major factors driving the growth besides increasing enforcement of health and safety regulations by governments and continuous development of ever cheaper and miniaturized gas sensors, is also the raised awareness of indoor and outdoor air quality control among the general population.

The single most significant obstacle on the road towards a more widespread use of gas sensors is their price. The cost of the majority of gas sensors on the market today is in the range of a dozen euros. However, almost exclusively, these sensors, which are discrete components, are embedded in instruments that bring the price up to an excess of hundreds or even thousands of euros for some highly sophisticated instruments like electronic noses. This greatly limits their application areas.

The future gas-sensor market expansion will most likely come through unit cost reduction achieved by further sensor miniaturization and co-integration with signal drive and conditioning, as well as processing circuitry. Such solid-state gas sensors [169] integrated alongside standard low-cost manufacturing technology (for example, CMOS) flow with price tags which will be as low as couple of euros per piece including read-out interfaces [163] would open several high-volume markets. Namely, towards the end of the next decade, gas sensors could potentially be included on a regular basis into many consumer electronic products such as laptops, tablets, cell phones, and fitness/smart watches whereas some technological difficulties like preserving waterproof design should be solved beforehand, at least in watches and cell phones. In this aspect, they could follow related success stories of low-cost temperature, barometric pressure, and/or humidity sensors, which all incorporate the sensing element together with surrounding electronics.

Certainly, the cost factor is not the only impediment to fabricating a commercially successful sensor. Sensor characteristics are generally summarized as “4S”: **s**ensitivity, **s**electivity, **s**tability, and **s**peed of response and recovery. In addition to these metrics, the sensor size is also of paramount importance as is its power consumption, especially in mobile or wireless sensor network battery-powered applications, and other mass markets like the internet of things (IoT) or wearables. The last requirement is particularly challenging when one considers that for optimum performance, the majority of gas sensors must be heated by a separate heating element to operating temperatures which are well above the ambient. Notwithstanding, intermittent gas sensor operation is the usual method to relax this concern. One advanced solution is to design devices that combine gas sensing and self-power generation in a wearable gas sensor device, obliterating the need for a power source/battery [170]. One of the advantages of conducting polymers is room temperature operation, so application of this semiconducting material most probably functionalized with a noble metal, or as a hybrid or nanocomposite is a future direction for overcoming the high gas sensor operating temperature of current commercial metal oxide semiconducting sensors [31,32,128].

**Figure 8:**
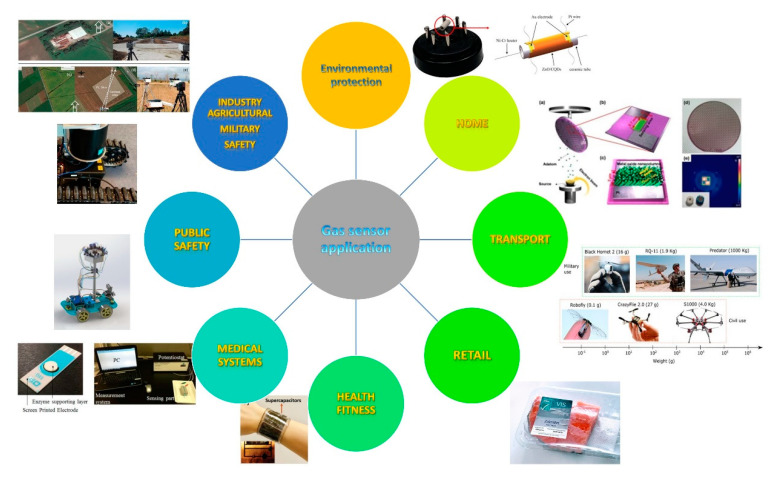
Scheme showing semiconductor gas-sensor applications, adapted from [170,171,172,173,174,175,176,177,178].

Apart from single dedicated gas sensors, there is a demand for multi-gas sensing systems/sensor arrays with roughly speaking a dozen of sensors (which may also include ambient temperature, humidity and barometric pressure). Both, individual gas sensors and gas sensor arrays, nowadays are in pervasive use in a variety of spheres. The following subsections describe the application areas of all types of gas sensors, with an overview of the current and research into future applications of semiconducting gas sensors, as shown in Figure 8.

### 5.1. Electronic Nose (e-Nose or eNose)

A gas sensor array in which every individual gas sensor is really not specific to a particular gas, but has a rather different sensing response pattern instead, is the basis for the fundamental sensing system commonly known as an “electronic nose”. When combined with a pattern recognition module it is considered a general olfaction structure for odor characterization [179]. The same sensors deployed in single gas applications are commonly used in electronic noses.

An electronic nose typically consists of a multisensor array, information-processing unit, software implementing pattern-recognition algorithms, and digital reference-library database of aroma fingerprint signatures. These systems can be used in different application areas requiring the sensing of a wide spectrum of gases.

Even though different types of gas sensor are employed in e-nose systems, semiconductor-based gas sensors are preferred primarily thanks to their fast response and recovery times [18], but also because their small size, low manufacturing cost, and acceptable power consumption [180] even for handheld devices.

Examples of application of electronic noses [19] practically correspond to those of the gas sensors themselves and include (in no particular order):

- explosive and flammable material detection for public safety and welfare as well as for passenger and personnel security in airline transportation;
- perfume and cologne development and choice of fragrance additives as well as personal application product enhancement and consumer appeal in cosmetics;
- ingredient or product consistency confirmation for brand recognition and consumer fraud prevention as well as detecting off-flavors and characterizing taste and smell to determine contamination or ripeness or spoilage inside food and beverage quality control assessment;
- safe food supply and crop protection, crop ripeness, and preservation treatments for harvest timing and storage in agriculture;
- monitoring smell related processing and quality during food production [181];
- checking product characteristics and consistency for processing controls as well as aroma and flavor uniformity across products, but also toxic gas leak detection and fire alarms for the purposes of safety, security, and proper work conditions in any manufacturing industrial sector;
- pathogen identification and disease detection together with patient treatment selection and prognoses as well as checking nutrition status, organ failures, disease diagnoses, metabolic disorders, and general physiological conditions inside medical, healthcare, and clinical sectors;
- biological and chemical weapons and explosive materials detection in defense and military sectors;
- quality control of drug purity including formulation consistency and uniformity in product mixtures for the pharmaceutical industry.

### 5.2. Safety and Security

One of the main purposes of gas sensors is maintaining safety and security. Hydrogen gas sensors [182] are necessary in each sector of the emerging hydrogen economy, in which hydrogen gas is exploited as an energy carrier and as a chemical reactant. Recent research has involved investigating NiO as a p-type semiconducting material in the form of nanowires for hydrogen gas sensing, achieving promising results [47]. Focus has been also on resistive type hydrogen sensors based on ZnO nanostructures using atomic layer deposition (ALD), a fabrication method that is compatible with semiconductor technology (MEMS) [183].

In addition to hydrogen, many other hazardous and toxic gases should be kept under control. As mentioned, the demand and application of gas sensors in practice began with inflammable gas alarms [184] to protect people from fatal gas hazards such as exposure to poisonous gases and gas explosions or incomplete combustion accidents. Fire alarms usually combine a smoke and/or thermal detector together with a semiconductor gas sensor [185] and are literally found in every place where humans live, from residential buildings and hotels to schools and office space. Gas sensors are present in homes for monitoring flammable gas levels inside gas-fired boilers [186]. There has also been an increasing demand for workplace safety in coal mines or certain industries like petrochemical, to name a few. Actually, the success story of semiconductor gas sensors can be partially explained by their high sensitivity to a broad range of inflammable and toxic gas species and their simple detection principle relying on changes of electrical properties due to interaction with the gas,

Application of multiple gas sensors includes monitoring and measuring hydrogen, methane and carbon monoxide/dioxide, and oxygen.

Semiconducting metal oxide gas sensors can be used to construct multi-sensor units (marketed as a FireNose) to discover and discriminate between known and unknown gases in harsh and uncontrolled conditions [187] or mobile (rescue) robots for gas leak detection and localization [188], as well as toxic smoke real-time mapping which could serve as an aid to firefighter brigades and teams. The field of mobile robotic olfaction customarily considers gas source localization and gas distribution mapping as two of its main tasks.

Semiconductor gas sensors that can detect total VOC (TVOC) concentrations indoors are of fundamental importance. Recent research has focused on the development of a nanowire array comprised of CuO, NiO, and Cr_2_O_3_, all p-type semiconducting metal oxides for the detection of volatile organic compounds, such as [189].

Finally, a breathalyzer (a portmanteau of breath and analyzer), i.e., a portable handheld breath alcohol checker that detects ethanol vapor in human breath for preventing drunken driving is another example of applying semiconductor gas sensors for safety purposes. Interestingly, the very same semiconductor gas sensors are not only exploited inside a car ignition locking system but are also utilized inside a brewing process control.

### 5.3. Health Care and Medical

Detection of disease-related gases is drawing increasing attention for medical purposes. Exhaled human breath contains thousands of different volatile organic compounds (VOCs), which are usually measured at the sub-ppm level or even lower concentrations in healthy subjects. The attractiveness of VOC analysis comes from its non-invasive character and can be applied to any stage of life from early childhood to late adulthood. Even though the correlation between a certain disease and some VOCs has been known for more than a century, only modern instruments based on semiconductor gas sensors can give quantitative measures necessary for strict clinical practice. Independent clinical trials [190] have shown the possibility of using breath for detecting serious illnesses, such as different types of cancer, diabetes, Alzheimer’s and Parkinson’s disease, multiple sclerosis, tuberculosis, chronic kidney disease, among other. An additional burst to the interest in VOCs examination and analysis for medical purposes has been provided by the development and the diffusion of solid-state gas sensors [191]; therefore, in the foreseeable future, breath tests are expected to become just as common (if not more) as blood tests are today. Semiconducting gas sensors are applied to test some of these gases, such as acetone, hydrogen peroxide, volatile sulfur, nitric oxide, and ammonia [191]. However, the research is being conducted into constructing eNose systems based on commercial MOX sensors to detect low concentrations of VOCs in breath [192], further improved by temperature modulation, containing 8 analog and 10 digital commercial MOX sensors [193].

Specific breath biomarkers cannot only indicate a presence of some diseases, but can also reflect general physical condition. An example of such devices are portable acetone analyzers that measure the ketone level and calculate body fat burning rate just from exhaled breath. These pocket-sized accessories that monitor fat metabolism and levels of ketosis are in a widespread use in various diet and fitness programs. An example of the application of semiconducting materials in breath analysis are nanofibers of SnO_2_ functionalized with reduced graphene (rGO) that were evaluated for sensitive detection of acetone and hydrogen sulfide in exhaled human breath [194].

In addition to healthcare applications, gas sensors are also used in the fast developing wearable biosensors market. Wearable biosensors [195] are complex miniature devices that incorporate wireless communication modules for transmitting sensor data to computing infrastructure. A variety of substances is used in such sensing devices. As an illustration, for semiconducting oxide materials, this is a challenge, as it is necessary to reduce the sensor operating conditions to room temperature. One way to achieve this is through nanostructures, like graphene [196] or nanowires. Recently, even a flexible and stretchable self-heating metal oxide (MOx) gas-sensing platform has been demonstrated [197].

Compared to their industrial counterparts, the development of wearable gas sensors needs to address additional challenging requirements, including a lightweight and small form factor, low operating temperature, low energy consumption, and mechanical robustness upon various skin deformations.

### 5.4. Air Quality and Environmental Monitoring

Pollution and urban air quality are major environmental risks [198] to public health. Gas emissions are responsible for a variety of respiratory illnesses and environmental problems, such as acid rain and depletion of the ozone layer. Pollutants may be released from natural sources and they can be synthetic or anthropogenic. Natural sources of air pollutants are lightning, soil, fires, and volcanoes while anthropogenic sources incorporate emissions from human activity—for example, exhaust gases from transportation, chemical accidents, or industrial actions such as power plants and landfill sites. Many countries express air quality in terms of the air quality index, which is calculated based on concentrations of several key air pollutants [199] such as ground-level ozone, particulates, sulfur dioxide, carbon monoxide, and nitrogen dioxide. Some of these are powerful oxidizing toxic gases that have noxious effects on both vegetation and human health. Ozone, as a secondary pollutant, for example, has become of growing importance in ambient air during the last decades [200] and has been identified as the main agent responsible for heavy peaks of pollution in urban atmospheres during warm sunny periods.

Commonly, data on air quality are collected from monitoring stations, which contain a sensor for each pollutant or a gas sensor array [201], just like in an electronic nose. Currently, many monitoring systems consist of a static network of air quality sensors that are distributed at key locations and can produce a spatially resolved picture of pollution variations on the urban scale. Consequently, in order to obtain a truthful representation of the gas distribution and be able to locate gas sources, it is essential to collect spatially distributed gas concentration data.

Mobile robots equipped with gas detectors are also used in outdoor applications for pollution monitoring and source localization in public areas, surveillance of industrial facilities producing harmful gases, and monitoring of landfill sites. Enhanced sensitivity to acetone and ethanol was achieved by a mobile robot system with a new signal processing technique [178]. A portable electronic nose featuring on-demand operation [202] is particularly neat for the IoT and wireless sensor networks.

Gas monitoring outdoors can also be addressed using unmanned aerial vehicles (UAVs), commonly known as drones, although with far more power and size constraint [203] limitations placed upon gas sensors. In addition to air quality and environmental monitoring in general [204], an individual and a swarm of micro and nanodrones [174] have also been employed for volcanic gas sampling, localization of fugitive emissions, early fire detection, precision agriculture, landfill monitoring, and mine blasting, among many other use cases.

The tiny form-factor and maneuverability of UAVs allow sensing of hazardous environments inaccessible to terrestrial robots. Miniaturization of semiconductor gas sensors made possible equipping drones with olfaction capabilities, which can be exploited in a myriad of applications. For example, in the aftermath of an earthquake or explosion drones could navigate such scenarios much faster and sample the space in 3D.

Both indoor and outdoor air quality concerns are driving the applications not only in environmental monitoring but in commercial building automation too. Therefore, gas sensors are experiencing a high demand in heating, ventilation, and air conditioning (HVAC) control systems as they facilitate intelligent ventilation control based on gas concentrations and this is one of the fields of commercial application of semiconducting gas sensors [205].

Furthermore, air quality sensors are usually incorporated into air cleaners, deodorizers, ionizers, purifiers, and air sanitizers for energy saving purposes or just as a plain amenity.

### 5.5. Automotive

Modern cars are abundant in gas sensors located both inside and outside the cabin serving as either a commodity or an essential indispensable part in the engine compartment.

It is nowadays common to have the electronic control unit (ECU) of a car automatically close and open the fresh air flaps [206], depending on the ambient gas concentration measured in the air-intake manifold under the hood of the car. Typical gases that are monitored are combustion-related compounds. Therefore, a pair of CO and NO_2_ sensitive sensors are regularly installed into a damper system to determine the “outside air quality level”. In addition, the car interior could also exhibit multiple sources of disturbing gases like cigarette smoke, food odor, or bio-effluents, which are detected by a sensor system directly mounted within the cabin of a passenger vehicle to define the “inside air quality level” thus requiring more sophisticated air and climate-management concepts. This means that based on interior and exterior semiconductor gas sensor readings, contemporary automotive heating, ventilation, and air conditioning (HVAC) systems can decide to take no action, close the recirculation flap in case of bad outside air quality level, increase air exchange rate in case of bad inside air quality level, or start active air cleaning. These automotive gas sensors are often metal oxide based [205]. One such metal oxide based sensor commercially produced, is advertised as the “smallest selective gas sensor” suitable for air quality monitoring in automotive, aerospace, and transportation applications [207].

### 5.6. Industrial

Applications of semiconductor gas sensors in industry are virtually countless and span across every industrial branch.

Mining and in particular, underground mining exposes workers to flammable gas, asphyxiates, oxygen depletion and a range of toxic gas hazards. To keep miners safe, both fixed and portable detectors are deployed to ensure that if a dangerous condition arises, audible and visual alarms are generated so that evacuation can occur rapidly. Likewise, in the gas and oil industry, whether upstream in the production process (exploration and extraction) or downstream (transportation, processing, storage) or around the distribution pipelines, many different gas detectors are used for process control as well as for staff and plant protection against explosion or the presence of toxic gases. Gas sensors are also used at rig and processing locations to monitor the concentration of the gases released into the Earth’s atmosphere. Similar use cases of gas sensors for various process control and safety of the employed personnel are encountered in petrochemical industry too. Though MOX sensors are not used in safety products to detect explosive gases, they are used to detect gas leaks and set off alarms warning of an explosive gas mixture, such as [208]. It is true that one of the disadvantages of metal oxide semiconducting gas sensors is their cross-sensitivity that can result in many false alarms, but if used as an eNose this problem can be overcome to some extent, especially taking into account the high sensitivity and low cost of these sensors [205].

Apart from toxic and explosive gas detection for the purpose of domestic, industrial, and public safety, semiconductor gas sensors are used in beverage and food industries to control the fermentation processes. A very interesting application is in intelligent food packaging [209] used to detect rotten or spoiled food using cheap gas sensors. For instance, these sensors can identify spoilage gases like ammonia in meat and fish products and can be read by smartphones. Even though the concept of food quality control [210] using gas sensors is already known for a quarter of a century, it has become economically feasible only recently. Eventually, these low-cost gas detectors costing about one cent might eventually replace the “use-by” date stickers, as a much more reliable freshness indicator. A more advanced multisensor array inside an electronic nose has been applied to determine optimal beef aging time [211] and monitor meat quality as well as to control food in general. The intelligent packaging concept using gas sensors as essential components means to enable monitoring and providing information on the condition of food, packaging, and the surrounding environment [212,213]. It is an essential component of smart food packaging. Therefore, application of gas sensors in the food production industry and packaging could greatly contribute to reducing food waste, but more importantly cut the yearly number of food poisonings contributing significantly to food safety.
